# Gender-Specific Differences in Low-Dose Haloperidol Response for Prevention of Postoperative Nausea and Vomiting: A Register-Based Cohort Study

**DOI:** 10.1371/journal.pone.0146746

**Published:** 2016-01-11

**Authors:** Florian Brettner, Silke Janitza, Kathrin Prüll, Ernst Weninger, Ulrich Mansmann, Helmut Küchenhoff, Alexander Jovanovic, Bernhard Pollwein, Daniel Chappell, Bernhard Zwissler, Vera von Dossow

**Affiliations:** 1 Department of Anaesthesiology, University Hospital of Munich, Munich, Germany; 2 Department of Medical Informatics, Biometry and Epidemiology, Ludwig-Maximilians-Universität München, Munich, Germany; 3 Statistical Consulting Unit Department of Statistics, Ludwig-Maximilians-Universität München, Munich, Germany; 4 Anaesthesia and Intensive Care Medicine Clinic, Klinikum Dritter Orden, Munich, Germany; University Hospital Llandough, UNITED KINGDOM

## Abstract

**Background:**

Postoperative nausea and vomiting (PONV) is one of the most common and distressing complications after general anesthesia and surgery, with young non-smoking females receiving postoperative opioids being high-risk patients. This register-based study aims to evaluate the effect of low-dose haloperidol (0.5 mg intravenously) directly after induction of general anesthesia to reduce the incidence of PONV in the postoperative anesthesiological care unit (PACU).

**Methods:**

Multivariable regression models were used to investigate the association between low-dose haloperidol and the occurrence of PONV using a patient registry containing 2,617 surgical procedures carried out at an university hospital.

**Results:**

Haloperidol 0.5 mg is associated with a reduced risk of PONV in the total collective (adjusted odds ratio = 0.75, 95% confidence interval: [0.56, 0.99], p = 0.05). The results indicate that there is a reduced risk in male patients (adjusted odds ratio = 0.45, 95% confidence interval: [0.28, 0.73], p = 0.001) if a dose of 0.5 mg haloperidol was administered while there seems to be no effect in females (adjusted odds ratio = 1.02, 95% confidence interval: [0.71, 1.46], p = 0.93). Currently known risk factors for PONV such as female gender, duration of anesthesia and the use of opioids were confirmed in our analysis.

**Conclusion:**

This study suggests that low-dose haloperidol has an antiemetic effect in male patients but has no effect in female patients. A confirmation of the gender-specific effects we have observed in this register-based cohort study might have major implications on clinical daily routine.

## Introduction

Postoperative nausea and vomiting (PONV) is a common and distressing complication following anesthesia and surgery and can result in dehydration, electrolyte imbalance, wound dehiscence, pulmonary aspiration and delayed hospital discharge [1, 2]. PONV is considered by many patients to be more distressing than surgical pain [3, 4]. The risk of developing PONV without pharmacological prophylaxis varies from about 20–30% overall to 70% depending on the patients’ baseline risk factors [5–8] and is a substantial economic factor [8]. In the last decade, several risk scores and pharmacological strategies have been evaluated to reduce the probability of suffering from PONV [9–13]. One of the pharmacological strategies to reduce PONV is the application of haloperidol, a neuroleptic drug with a further indication as an antiemetic substance [13–15]. Several studies investigated its effectiveness on preventing PONV. Most of the studies were performed with doses of at least 1 mg haloperidol intravenously (i.v.) or in combination with other antiemetic drugs like dexamethasone or ondansetron [16–20]. To our best knowledge, there are no data available demonstrating gender effects of haloperidol used as an antiemetic drug.

Following the Food and Drug Administration (FDA) warning for droperidol, haloperidol was established in our hospital as one prophylactic medication against PONV. Since side effects such as dystonia or tardive dykinesia were observed when using higher dosages, e.g., 2 mg, the current standard used in our hospital for PONV prophylaxis is 0.5 mg haloperidol.

This register-based retrospective cohort study aimed to evaluate the effect of low-dose haloperidol (0.5 mg i.v.) directly after the induction of general anesthesia for the prevention of PONV in the postoperative anesthesiological care unit (PACU), while adjusting for confounders like gender, age, BMI, smoking status, ASA status (preoperative classification of the American Society of Anaesthesiologists), type and duration of anesthesia and use of opioids. The structure of this paper follows the STROBE guidelines for reporting observational studies.

## Methods

The study was approved by the Ethics Committee of the Ludwig-Maximilians-University Munich (no. 042–13, accepted 01/30/2013). The Ethics Committee waived the requirement for informed consent from the participants in the study. We obtained data from a patient registry of the Department of Anaesthesiology at the University Hospital of Munich in which information on all anesthesiological procedures is routinely electronically documented. Patient data was anonymized and de-identified prior to access and analysis. It is available as S1 Dataset. The data has not been considered for any other study.

This register-based cohort study comprised a group of patients having received a dose of 0.5 mg haloperidol i.v. (haloperidol group) and a group of patients not having received any antiemetic drugs for prevention of PONV (non-haloperidol group). Due to a long elimination half-life time (about 15 h) and a potential intraoperative initiation of the antiemetic effect, haloperidol was applicated directly after induction of general anaesthesia [21].

Information on patient characteristics and surgical details were retrieved from a patient registry where data were documented according to a standard protocol. Each anesthesiological procedure at the University Hospital of Munich is routinely recorded using the online documentation software NarkoData^®^ (Ver. 4.7.0.3, IMESO GmbH, Giessen, Germany).

All patients receiving general anesthesia with successive surgery within the period 01/07/2008–06/19/2012 at the University Hospital of Munich were considered for this study. The inclusion criteria were: age of at least 18 years and having received a surgical procedure in the domain of urology, gynecology or general surgery (including laparotomy and opening of the retroperitoneal space, operations at the kidney, cystectomy, replacement of the urinary bladder, other operations at the urinary organs, excision and destruction of prostate tissue, radical prostatectomy, local excision and destruction of ovarial tissue, oophorectomy, hysterectomy, partially excision of the mammarian glandula and destruction of mammarian tissue, excision and resection of the mammarian glandula). Patients who received a different dose than 0.5 mg haloperidol were excluded as well as patients who received other antiemetic drugs such as ondansetron before or during anesthesia.

Early PONV was defined if patients received ondansetron as rescue treatment antiemetic drug *after* the surgical procedure in the recovery room (according to our clinical standard). In this context “early” refers to the time period from extubation until discharge from the PACU [22]. Ondansetron was only used as a therapeutic drug for the treatment of PONV in the PACU. Patients received either no haloperidol (non-haloperidol group) or a dose of 0.5 mg haloperidol applied intravenously directly after induction of general anesthesia (haloperidol group).

Due to a low number of ASA 4 patients, ASA 3 and 4 patients were pooled into one category ASA 3–4.

Data concerning intravenous drugs, epidural drugs and preoperative patient data (e.g. history of PONV, smoking status) were documented manually during the surgical procedure by the treating anaesthesiologist. Data concerning the volatile anaesthetics (maximum and average concentrations) were documented automatically online by the anesthesia documentation software NarkoData.

Patient characteristics were entered from a standard paper-based protocol in a database (single data entry). Consistency checks were done before performing the statistical analysis.

The number of surgical procedures that fulfilled the inclusion and exclusion criteria and were conducted within the period 01/07/2008–06/19/2012 at the University Hospital of Munich determined the sample size.

In observational studies the treatment is not randomized within the patient population. Therefore, systematic differences in the characteristics of the treatment and the control group can occur. In order to avoid biased treatment effect estimates appropriate statistical analyses such as multivariable regression or propensity score analyses have to be used to address this problem [23, 24]. We applied both procedures to account for the problem of systematic differences between the haloperidol and the non-haloperidol group.

To assess the patients’ baseline risks for PONV, the Apfel score was computed which is a simplified risk score for predicting the patient’s risk for PONV within 24h of surgery [12, 25]. It corresponds to the number of risk factors out of the following factors: female gender, history of PONV, non-smoking and the use of postoperative opioids (here: piritramide). Apfel scores of 0, 1, 2, 3, 4 correspond to PONV risks of 10%, 20%, 40%, 60% and 80%.

A chi-square test was performed for testing whether PONV incidences differed between the haloperidol and the non-haloperidol group. A Mann-Whitney-U test was used for testing whether duration of stay in the recovery room was different between patients with PONV and patients without PONV.

A logistic additive model with the outcome “occurrence of PONV” was used. Besides the variable indicating if the patient had received haloperidol, our model included all the confounder variables which were known from the literature and were documented in the patient registry, namely: gender, age, BMI, smoking status, previous PONV, type of surgical procedure, combination anesthesia (general plus epidural anesthesia), duration of anesthesia, dose of sufentanil, remifentanil, piritramide, sevoflurane and propofol. No preceding variable selection was performed. The effects of all metric covariates in the additive model were modeled by smooth functions, that allow for a flexible modelling. Tuning parameters that control the wiggliness of the curves were adjusted via generalized cross-validation implemented in the R package mgcv [23]. 95% pointwise confidence limits were computed that indicate whether the odds ratio (OR) at a given predictor value significantly differs from 1. This is the case if the confidence interval (CI) for the log OR does not cover the value zero. Apart from all main effects an interaction effect between haloperidol and gender was included into the model to allow for the estimation of gender-specific associations between haloperidol and PONV (the decision of including the interaction was made based on a post-hoc analysis).

In a secondary analysis, we used propensity score methods for confounder adjustment in the population of female and male patients to see if results from propensity score analysis are consistent with those obtained from multivariable regression.

In a second model the duration of stay in the recovery room for patients having PONV was compared to the duration of stay for patients not having PONV while adjusting for the confounder variables gender, age, BMI, smoking status, history of PONV, type of surgical procedure, combined anesthesia, haloperidol for prophylaxis of PONV, dose of sufentanil, remifentanil, piritramide, sevoflurane and propofol. A linear additive regression model was used that includes all main effects and the interaction between haloperidol and gender. A logarithmic transformation was performed on the response variable “duration of stay”.

Statistical analysis was performed using the statistical software R (version 3.0.1).

## Results

**Fig 1:**
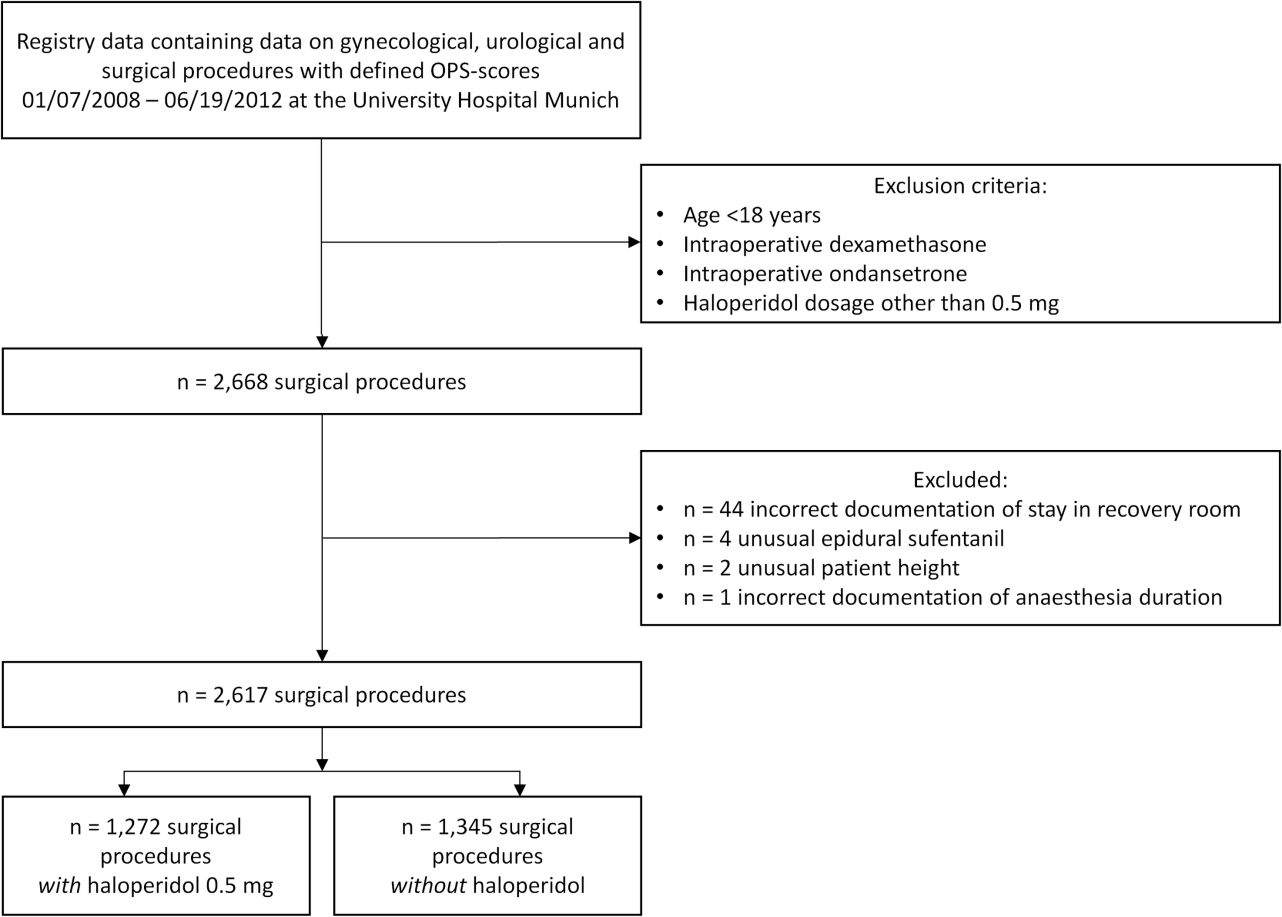
Enrollment. Enrollment of surgical procedures into the analysis.

A total of 2,668 surgical procedures documented in the patient registry fulfilled the inclusion criteria (Fig 1). Information on 51 surgical procedures was excluded from analysis due to incorrect documentation (see Fig 1). Data from 2,617 surgical procedures remained for statistical analysis.

Patient characteristics are shown in Table 1. In 1,272 of the 2,617 surgical procedures (48.6%) haloperidol was administered, while in the remaining 1,345 no prophylactic antiemetic substance was administered. Pronounced differences between the haloperidol and the non-haloperidol group were seen for gender, ASA status, surgical procedures, age, kind of anesthesia, anesthetic drug characteristics. This illustrates the need for appropriate statistical methods to adjust for differences between the groups.

**Table 1:** Patient characteristics.

Variable			Overall (n = 2,617)	Haloperidol group (n = 1,272)	*Non-haloperidol group (n = 1*,*345)*
***Gender***					
	Male		52.7%	41.1%	63.6%
	Female		47.3%	58.9%	36.4%
***Smoking status***					
	Smoker		15.6%	14.5%	16.6%
	Non-smoker or unknown		84.4%	85.5%	83.4%
***History of PONV***					
	Positive		2.5%	2.4%	2.5%
	Negative or unknown		97.6%	97.6%	97.5%
***ASA***					
	*ASA 1*		10.9%	15.3%	6.8%
	ASA 2		52.1%	59.4%	45.3%
	ASA 3–4		37.0%	25.4%	48.0%
***Surgical procedure***					
	Laparotomy and opening of the retroperitoneal space		29.1%	18.0%	39.6%
	Operations at the kidney		17.5%	12.6%	22.2%
	Cystectomy		1.4%	2.1%	0.7%
	Replacement of the urinary bladder		3.7%	3.9%	3.4%
	Other operations at the urinary organs		11.7%	12.3%	11.0%
	Excision and destruction of prostate tissue		8.0%	7.4%	8.6%
	Radical prostatectomy		7.8%	8.0%	7.5%
	Local excision and destruction of ovarial tissue		3.3%	6.1%	0.7%
	Oophorectomy		1.3%	2.0%	0.6%
	Hysterectomy		7.2%	12.2%	2.5%
	Partial excision of the mammarian glandula and destruction of mammarian tissue		5.5%	9.0%	2.1%
	Excision and resection of the mammarian glandula		3.6%	6.3%	1.1%
***Combined anesthesia***					
	Yes		46.5%	44.3%	48.6%
	No		53.5%	55.7%	51.5%
***Age [years]***			60.9 ± 13.4	60.0 ± 13.7	61.7 ± 13.0
***BMI [kg/m***^***2***^***]***			26.3 ± 4.8	26.4 ± 4.8	26.2 ± 4.9
***Anesthesia duration [min]***			184.0 ± 89.0	194.7 ± 94.7	173.9 ± 82.0
***Sufentanil***					
	Epidural (bolus)				
		0 μg	56.7%	57.6%	55.8%
		10 μg	42.6%	41.5%	43.6%
		20 μg	0.7%	0.9%	0.6%
	Intravenous (bolus), [μg]		44.6 ± 21.3 ^a^; n = 1979 ^a^	45.4 ± 20.0 ^a^; n = 910 ^a^	43.9 ± 22.3 ^a^; n = 1069 ^a^
	Intravenous (infusion, peak rate), [μg/h]		42.7 ± 17.1 ^b^; n = 26 ^b^	47.3 ± 20.2 ^b^; n = 15 ^b^	36.4 ± 9.2 ^b^; n = 11 ^b^
	Intravenous (TCI, total dose), [μg]		74.6 ± 38.5 ^c^; n = 71 ^c^	71.1 ± 24.9 ^c^; n = 55 ^c^	86.6 ± 67.0 ^c^; n = 16 ^c^
***Remifentanil***					
	Intravenous (bolus), [μg]		95.4 ± 47.7 ^d^; n = 13 ^d^	94.3 ± 32.1 ^d^; n = 7 ^d^	96.7 ± 65.0 ^d^; n = 6 ^d^
	Intravenous (infusion, peak rate), [μg/h]		755.4 ± 372.6 ^e^; n = 579 ^e^	778.6 ± 390.6 ^e^; n = 310 ^e^	728.7 ± 349.5 ^e^; n = 269 ^e^
	Intravenous (TCI, total dose), [μg]		1,473.1 ± 1,003.3 ^f^; n = 468 ^f^	1,523.9 ± 1,076.0 ^f^; n = 295 ^f^	1,386.6 ± 861.6 ^f^; n = 173 ^f^
***Piritramide***					
	Intravenous (bolus), [mg]		10.7 ± 7.2 ^g^; n = 778 ^g^	9.1 ± 5.9 ^g^; n = 373 ^g^	12.1 ± 7.9 ^g^; n = 405 ^g^
***Sevoflurane***					
	Max. conc. [Vol%_et_]		1.84 ± 0.76 ^h^; n = 499 ^h^	1.84 ± 0.82 ^h^; n = 195 ^h^	1.84 ± 0.72 ^h^; n = 304 ^h^
	Average conc. [Vol%_et_]		0.93 ± 0.65 ^i^; n = 544 ^i^	0.92 ± 0.68 ^i^; n = 212 ^i^	0.94 ± 0.64 ^i^; n = 332 ^i^
***Propofol***					
	Intravenous (bolus), [mg]		198.1 ± 53.6 ^k^; n = 1,902 ^k^	197.5^l^ ± 51.1 ^k^; n = 845 ^k^	198.6 ± 55.5 ^k^; n = 1,057 ^k^
	Intravenous (infusion, peak rate), [mg/h]		441.3 ± 100.7 ^l^; n = 1,797 ^l^	440.0 ± 91.9 ^l^; n = 811 ^l^	442.3 ± 107.4 ^l^; n = 986 ^l^
	Intravenous (TCI, total dose), [μg]		1,450.1 ± 749.4 ^m^; n = 580 ^m^	1,471.5 ± 745.9 ^m^; n = 382 ^m^	1,408.8 ± 756.3 ^m^; n = 198 ^m^

Patient characteristics documented for the 2,617 surgical procedures that were used for the statistical analysis overall, in the subgroup of patients having received haloperidol (haloperidol group) and in the subgroup of patients having not received any antiemetic substance (non-haloperidol group). For categorical variables percentages are given, for continuous variables the mean value ± standard deviation is given.

^a^ Sufentanil bolus > 1μg

^b^ Sufentanil continuous rate ≥ 10mg/h

^c^ Sufentanil TCI total dose > 1μg

^d^ Remifentanil i.v. > 0μg

^e^ Remifentanil continuous rate > 0μg/h

^f^ Remifentanil TCI total dose > 10μg

^g^ Piritramide dosage > 0mg

^h^ maximum endtidal sevoflurane concentration > 0.3%

^i^ Average sevoflurane concentration > 0

^k^ Propofol i.v. bolus > 0mg

^l^ Propofol continuous peak rate > 20mg/h

^m^ Propofol TCI total dose > 1mg. Sufentantil and remifentanil were given only intraoperatively, whereas piritramide was applicated only in the PACU.

**Fig 2:**
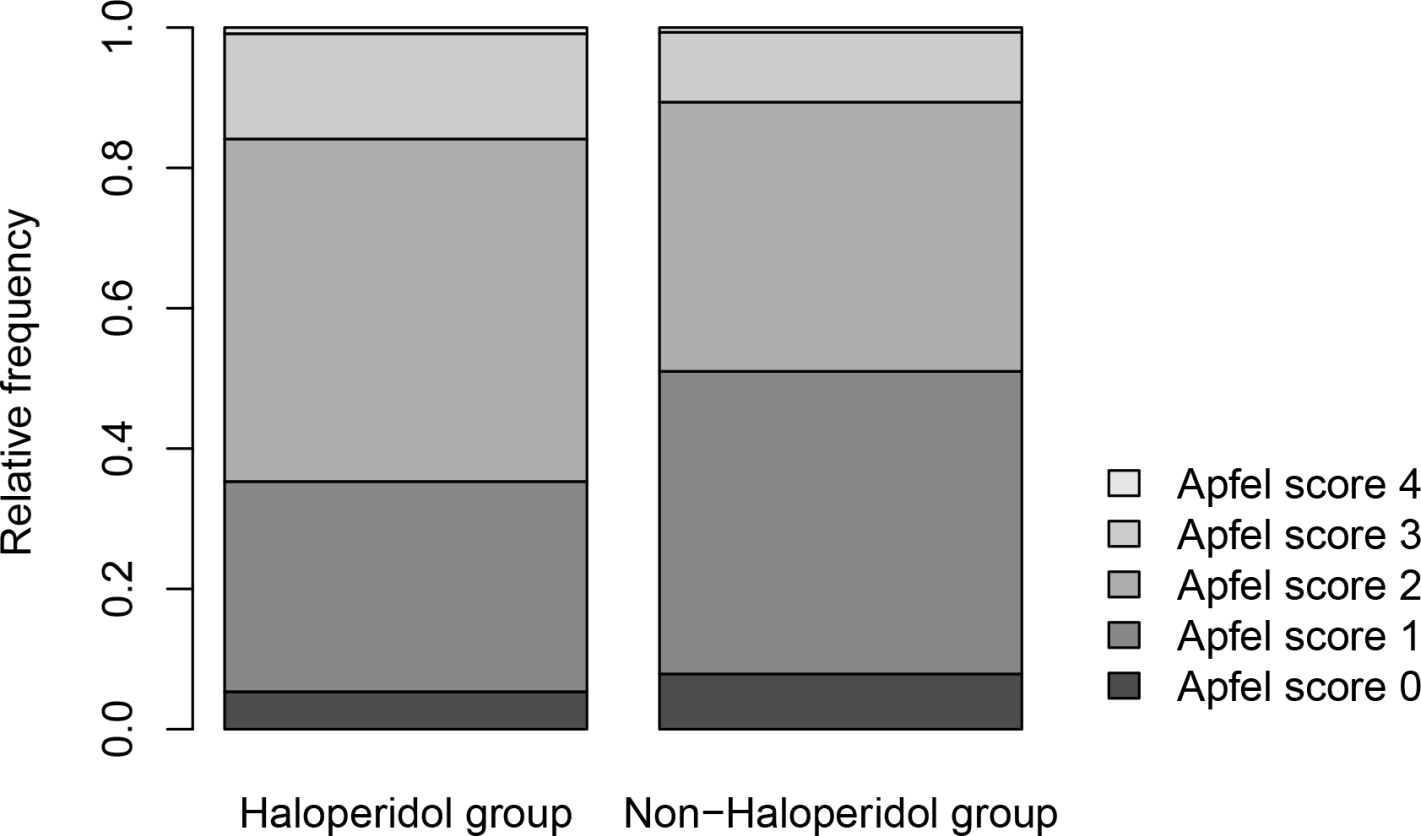
Apfel scores. Relative frequency of patients with Apfel scores 0–4 in the haloperidol group and in the non-haloperidol group.

Fig 2 shows the baseline risks for PONV in the haloperidol group and in the non-haloperidol group according to the Apfel score. There is a preferential application of haloperidol for patients with higher Apfel scores. For example in the non-haloperidol group approximately 50% of the patients have an Apfel score of 0 or 1, while in the haloperidol group only about 35% have an Apfel score equal or less than 1. This underlines the need for risk factor adjustments when assessing the use of haloperidol for the prevention of PONV.

In total 338 patients (12.9%) suffered early PONV (i.e., PONV in PACU). The average length of stay in PACU was 150±83 min. There were no differences in the incidence of PONV between the non-haloperidol group (n = 178; 13.2%) and the haloperidol group (n = 160; 12.6%), giving an unadjusted odds ratio of 0.94 (95% CI: [0.75, 1.19]) in the total patient collective (p = 0.66). In the female population 18.5% of the patients had PONV and 7.9% had PONV in the male population. The unadjusted odds ratio for the effect of haloperiol in the subgroup of females and males, respectively, was 0.87 (95% CI: [0.65, 1.16], p = 0.37) and 0.54 (95% CI: [0.35, 0.84], p = 0.008).

The multivariable regression model confirmed risk factors for PONV, which are reported in the literature. These factors determine subgroups of patients with a specific PONV risk. The PONV risk spectrum for male patients when not treated with haloperidol ranges from 0 to 57%. When treated with haloperidol each male subgroup risk changes by the OR of 0.45 (95% CI for the OR: [0.28, 0.73], p = 0.001). The PONV risk spectrum for female patients when not treated with haloperidol ranges from 0 to 86%. For female patients the adjusted OR is close to 1, which corresponds to no change in PONV risk when haloperidol is administered.

When not integrating an interaction between gender and haloperidol, an adjusted OR of 0.75 was obtained for the total patient collective (p = 0.05).

**Fig 3:**
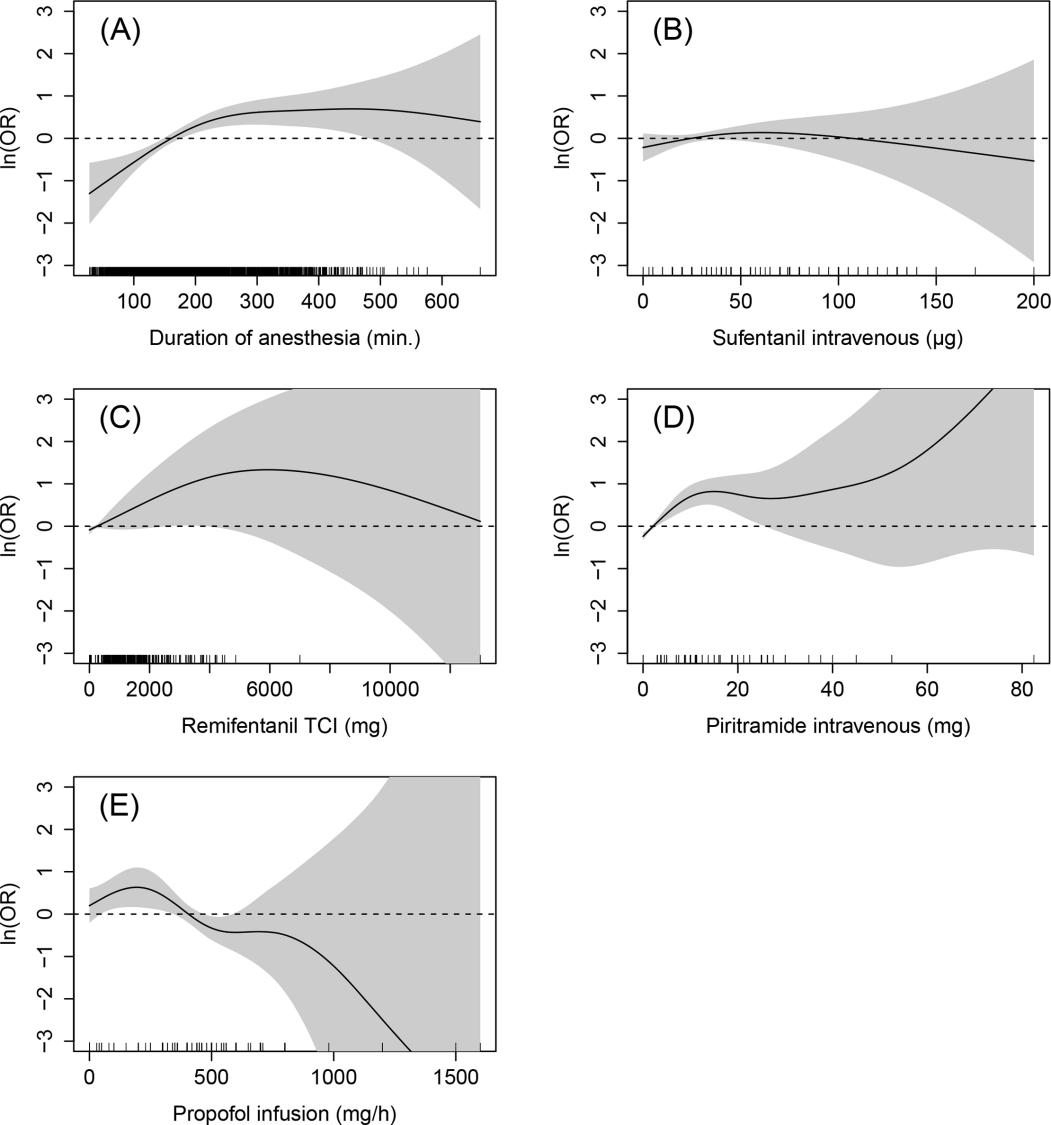
Nonlinear covariate effects. Estimated nonlinear effects of anesthesia duration (A), intravenous sufentanil (B), remifentanil TCI total dosage (C), piritramide intravenous (D) and propofol infusion maximum rate (E). The solid black line visualizes the estimated effect (measured on log OR scale), the grey region corresponds to the 95% pointwise confidence interval for the estimated effect. The marks along the x-axis indicate the observed covariate values in the data.

The adjusted ORs and 95% CI for the confounding variables estimated via the multivariable regression model are shown in Table 2. For metric variables an OR is provided if the effect is nearly linear, otherwise the effect (in terms of the log OR) is described via a smooth curve (Fig 3). The effects were non-linear for the duration of anesthesia, total intravenous sufentanil, remifentanil and piritramide application as well as for the maximum continuous rate of propofol.

**Table 2:** Adjusted Odds Ratios.

Variable			OR	95% CI	p-value
***Haloperidol–male***			0.45	[0.28, 0.73]	0.001
***Haloperidol–female***			1.02	[0.71, 1.46]	0.93
***Interaction*: *haloperidol*, *female***			2.25	[1.25, 4.05]	0.007
***Gender (reference*: *male)***			2.66	[1.80, 3.93]	<0.001
***Smoking status (reference*: *non-smoker)***			0.78	[0.54, 1.13]	0.20
***History of PONV (reference*: *negative)***			1.59	[0.82, 3.05]	0.17
***ASA (reference*: *ASA 1)***					0.001^a^
	ASA 2		0.56	[0.37, 0.86]	0.008
	ASA 3–4		0.41	[0.25, 0.66]	<0.001
***Surgical procedure (reference*: *Laparotomy and opening of the retroperitoneal space)***					<0.001^a^
	Operations at the kidney		0.74	[0.51, 1.06]	0.10
	Cystectomy		1.09	[0.41, 2.94]	0.86
	Replacement of the urinary bladder		0.71	[0.35, 1.44]	0.34
	Other operations at the urinary organs		0.10	[0.02, 0.45]	0.002
	Excision and destruction of prostate tissue		1.68	[0.97, 2.91]	0.06
	Radical prostatectomy		0.64	[0.32, 1.26]	0.19
	Local excision and destruction of ovarial tissue		0.25	[0.09, 0.70]	0.008
	Oophorectomy		0.54	[0.15, 1.95]	0.35
	Hysterectomy		0.73	[0.46, 1.17]	0.19
	Partial excision of the mammarian glandula and destruction of mammarian tissue		0.50	[0.23, 1.06]	0.07
	Excision and resection of the mammarian glandula		0.40	[0.17, 0.94]	0.04
***Combined anesthesia***			1.12	[0.52, 2.41]	0.77
***Age (+ 10 years)***			1.00	[0.90, 1.11]	0.96
***BMI (+ 5 kg/m***^***2***^***)***			1.08	[0.95, 1.23]	0.23
***Anesthesia duration***			-	-	<0.001
***Sufentanil***					
	Intravenous (bolus, total dose)		-	-	0.36
	Epidural				0.03^a^
		10 μg vs. none	2.06	[1.02, 4.16]	0.04
		20 μg vs. none	5.21	[1.48, 18.36]	0.01
	Intravenous (infusion, continuous rate; + 25 μg)		0.92	[0.42, 1.99]	0.83
	Intravenous (TCI, total dose; + 10 μg)		1.10	[1.00, 1.21]	0.05
***Remifentanil***					
	Intravenous (bolus, + 100 μg)		0.79	[0.13, 4.89]	0.80
	Intravenous (infusion, continuous rate; + 100 μg)		1.01	[0.98, 1.05]	0.48
	Intravenous (TCI, total dose)		-	-	0.20
***Piritramide***					
	Intravenous (total dose)		-	-	<0.001
***Sevoflurane***					
	Average concentration (+ 0.1 Vol%_et_)		0.96	[0.90, 1.02]	0.15
	Max. concentration (+ 1 Vol%_et_)		1.52	[1.07, 2.16]	0.02
***Propofol***					
	Intravenous (bolus; + 50 mg)		0.96	[0.84, 1.08]	0.48
	Intravenous (infusion, continuous rate, mg/h)		-	-	0.03
	Intravenous (TCI, total dose; + 200 mg)		0.90	[0.84, 0.96]	0.003

Adjusted odds ratios (OR) with 95% confidence intervals (CI) and p-values (Wald test) computed from a multivariable logistic additive regression model which includes the interaction between haloperidol and gender.—indicates that the estimated effect is non-linear and can only be described via a graph (Fig 3).

^a^ p-value resulting from the Wald test for testing all levels of the respective covariate.

Vol%_et_ = Volume% endtidal.

There is a significant effect for gender indicating that female patients have an approx. 3-fold higher risk for PONV than male patients with similar characteristics (p<0.0001). No association was observed between age or BMI and the risk for PONV. A slightly higher but non-significant risk can also be seen for non-smokers compared to smokers (OR = 1/0.78 = 1.28, p = 0.20). Patients with positive history of PONV have a higher risk for PONV (OR = 1.59, p = 0.17) compared to patients who have never experienced PONV before or for whom no information regarding PONV history was available, though also this association is not statistically significant. The risk for PONV is reduced with increasing ASA status (p = 0.001). Patients with ASA 2 have a risk that is significantly lower at factor 0.56 (p = 0.008) and for patients with ASA 3 or 4 the risk is even lower at factor 0.41 (p<0.001), when compared to patients with ASA 1. Significant differences were also observed between different surgical procedures (p<0.001): patients receiving an operation at the urinary organs have the lowest risk for PONV, while those with excision and destruction of prostate tissue have the highest risk. The risk for latter patients is about 17 (= 1.68/0.1) times higher compared to patients with surgery on the urinary organs, indicating huge risk differences depending on the surgical procedure. ORs for other surgical procedures (when compared to laparotomy and opening of the retroperitoneal space) are shown in Table 2. Patients receiving combined anesthesia have almost the same risk for PONV as patients receiving general anesthesia alone (OR = 1.12, p = 0.77).

Fig 3(A) shows the non-linear effect of the duration of anesthesia on the risk of PONV. The black line corresponds to the estimated log OR, which depends on the specific values for duration of anesthesia. The grey regions are pointwise confidence intervals, and in this case are very narrow indicating a precise estimation of the log OR. Fig 3(A) shows a linear increase in the risk for PONV with increasing anesthesia durations up to 200 min; for durations above 200 min, the risk of PONV stays constantly high and does not further increase for higher durations. From this graphical representation one can for instance see that a patient with an anesthesia duration of 300 min has an about twice (= exp(0.65)) higher risk compared to a patient with an average duration of 184 min.

Regarding the administration of sufentanil, remifentanil and piritramide, the results indicate that the higher the total dose / peak rate, the higher the patient’s risk for developing PONV. Patients have a doubled risk for PONV when having received 10 μg sufentanil epidurally (p = 0.04). Increasing dosages of piritramide lead to an increasing risk of PONV (Fig 3(D)). With values above 15 mg, the effect does not further increase but stays constantly high. The effects of sufentanil (total bolus dose, infusion rate) and remifentanil (total bolus dose, infusion rate, target-controlled-infusion total dose) were not significant. For sufentanil (infusion rate) and remifentanil (bolus) this might possibly be attributable to the low statistical power to detect significant associations because of the small numbers of patients having received these opioids (n = 26 and n = 13, respectively; see Table 1). However, most of them have a trend towards an increased risk for higher values. A clear trend can for instance be observed for the effect of remifentanil when administered by target-controlled-infusion (TCI) of anesthesia (Fig 3(C)): for total doses below 5 g there is a nearly linear trend towards a higher risk of PONV.

There is also a significant increase in the risk for PONV for higher maximal concentrations of sevoflurane (p = 0.02): The risk for PONV is larger at factor 1.52 for an increase by 1 Volume% endtidal. However, there was no significant effect for the average concentration (p = 0.15).

For higher total doses of propofol applicated via TCI there is a linear decrease for the risk of PONV (p = 0.003). The OR for an increase of the total dose by 200 mg is 0.9. The effect of propofol infusion is shown in Fig 3(E) (p = 0.03). There is an initial slight increase in risk for values between 0 and 200 mg/h. For values above, there is a trend towards a reduced risk.

The results of propensity score analyses were in line with the results that were obtained from multiple regression. Marginal ORs (obtained using several different approaches) consistently indicated a substantially reduced risk for PONV in the male patient population and a constant or slightly reduced risk in the female patient population when receiving haloperidol (data not shown).

In the univariate analysis the average length of stay in the recovery room was 150±83 min overall, 200±87 min in the group of patients with PONV and 142±80 min in the group of patients without PONV in PACU (p<0.001). When adjusting for confounders by multivariable analysis an increased duration of stay in the recovery room by factor 1.31 (95% CI: [1.24, 1.39], p<0.0001) was observed for patients suffering from PONV.

## Discussion

Regression analyses on observational data are able to quantify associations between the outcome variable and covariates of interest. In this sense, a different association between the risk for PONV and low-dose haloperidol was detected in male and female patients from a register-based cohort study. Recent developments in causal statistics allow interpreting associations also in terms of effects. Especially the development of propensity scores strengthens this approach. Our additional analyses based on propensity score strategies agree with our traditional regression approach. Therefore, the data also indicates that low-dose haloperidol lowers the risk for PONV in male patients (OR = 0.45), while for female patients it shows no effect concerning the risk for PONV (OR = 1.02). With the use of generalized additive regression models we confirmed risk factors which were already known from the literature, such as female gender, ASA status, the use of inhalational anesthesia and opioids [12, 25, 26]. In the present study a significant difference in the risk for PONV depending on the type of surgical procedure being performed was detected (p<0.001).

In general, it is difficult to obtain unbiased treatment effects from observational studies as these are prone to bias and confounded by factors related to the treatment and the outcome. We applied multivariable regression as well as propensity score analyses in order to address this problem. To our best knowledge, this is the first study to investigate gender differences of the PONV-reducing effects of low-dose haloperidol in surgical patients. Most of the studies evaluated haloperidol dosages higher than 0.5 mg [27–30] or in a selected female collective [16, 29, 31]. Interestingly, several studies detected high effectiveness of haloperidol in reducing PONV in female patients [16, 20, 31, 32], whereas others only report a weak or no effect [16, 33]. Buettner and colleagues found in their meta-analysis lack of efficacy for 0.25 mg haloperidol and different numbers needed-to-treat in dependence of the dosage (0.5–4 mg) [14]. In the present study there was only evidence for a PONV lowering effect of 0.5 mg haloperidol in male patients, so that a gender effect of haloperidol could be assumed. Zhang-Wong and colleagues demonstrated that gender may possibly affect haloperidol metabolism at least after oral application [34]. In female rats haloperidol leads to a striatal dopamine efflux twice as large as in male rats. Moreover, Arenas and colleagues could demonstrate that gender-specific differences are dose dependent in studies investigating the effect of haloperidol on escape-avoidance in mice. They demonstrated a decreased effect in female animals in low dose applications [35–37], while there was no difference in effects between male and female animals when using higher doses of haloperidol. These findings suggest gender-specific differences also in humans when using small doses of haloperidol.

Potentially, a higher dosage could be effective in females as indicated by the many studies mentioned above. However, most of these studies do not report results for *early* PONV, which is considered in this paper. In the study by Chu et al. it was reported that PONV occurred within the first 2 hours in 8% of the female patients having received 2 mg Haloperidol intravenously and in 12% of the female control group (no statistically significant difference) [31]. In the study of Tornetta, an antiemetic effect of 0.5 mg for reducing PONV within 24 hours after surgery in females was shown [15]. However, we are not aware of any studies that evaluate the effect of 0.5 mg haloperidol on early PONV in female patients.

Similar to many other studies, in this study, haloperidol was administered immediately after induction of anesthesia. In a randomized double-blind trial it was shown that there was no difference in the risk for PONV for patients having received haloperidol during induction of anesthesia and patients having received haloperidol 30 min before the end of surgery [38]. Thus the results of our study are not affected by the timing of the administration of haloperidol.

In this paper, early PONV was diagnosed if patients received ondansetron as a rescue treatment antiemetic drug after the surgical procedure in the recovery room. However, no data was available from the patient registry which measures the severity of PONV. Some studies have also evaluated the severity of PONV based on absolute counts, Likert scales or visual analogue scales. Such scores could be used for a more detailed aquisition of PONV severity.

The mean duration in the recovery room until PACU discharge was 2 1/2 hours in our patient collective. Within this time period, a total of 338 patients (12.9%) in the non-haloperidol group had PONV. This number was much smaller than the number of patients with PONV within 24 hours of surgery which is predicted by the Apfel score (12.9% observed versus 33.1% predicted). The reason for this difference might be that many patients develop PONV in the delayed postoperative period. In the study of Chu et al. only about 20% of the patients who suffered from PONV within 24 hours after surgery, had PONV in the first two hours [15]. In other studies similar delayed and early PONV rates were observed [39]. As indicated by some studies the risk factors for early and delayed PONV differ, which might explain the differences in the occurrence of delayed and early PONV in these studies [40, 41]. Apfel et al., for example, showed that volatile anesthetics, such as sevoflurane, as well as its dosage are the strongest risk factor for early PONV, while it has less effect on the risk for delayed PONV. In our patient cohort only a small fraction of the patients received volatile anesthetics, which might be one reason for the small number of patients with early PONV [40].

Although this is a single-center study conducted at an university hospital, the results should be generalizable since the surgical procedures that were included are standard procedures and the respective patient populations should not differ from patients at smaller hospitals.

The most relevant limitation concerns the study design per se, which is purely observational. Although the registry contains all confounders reported in the literature, the possibility that unknown confounders exist which may bias our results cannot be excluded. Further studies, that are not affected by unmeasured confounders, such as randomized controlled trials, are necessary to validate our findings. Albeit our study does not give definitive evidence for a gender-specific effect of low-dose haloperiol, this finding, which is based on a comprehensive and carefully conducted analysis of a large patient cohort, deserves further investigations because if validated in future studies, it has an impact on clinical daily routine and reveals unexplored differences in mechanisms between men and women.

Besides the study design, the suspected incomplete documentation of smoking status and history of PONV might be considered as a problem. Compared with community prevalence of smoking status or PONV history our values seem to be too low for both parameters [6, 42, 43]. However, the imprecise documentation should not lead to systematic bias of effect estimates of any variable since the missing mechanism for smoking status and history of PONV should be completely at random. Instead, we expect the incomplete documentation to only affect the power of our analysis to detect a significant association between PONV and smoking status or between PONV and history of PONV, respectively. Our analysis indicated an increased risk for non-smokers and for patients with history of PONV, both being consistent with findings of other studies. However, these associations were not significant which we expect is due to the incomplete documentation and the resulting loss in statistical power.

The present study indicated a gender dependent effect of low-dose haloperidol concerning reduction of postoperative nausea and vomiting in male patients. There was no indication of risk reduction by the use of haloperidol in female patients. Our findings are based on an observational study. Further studies which are carefully conducted–preferably, randomized controlled trials, are needed to validate these findings.

## Supporting Information

S1 Dataset Data on 2,617 surgical procedures carried out at the University Hospital of Munich. (CSV)
